# 500. A Real-World Cohort Study of Bamlanivimab Versus Bamlanivimab-Etesevimab for Non-severe COVID-19

**DOI:** 10.1093/ofid/ofab466.699

**Published:** 2021-12-04

**Authors:** Lea Monday, George J Alangaden, Indira Brar, Ramesh Mayur

**Affiliations:** 1 Henry Ford Health System, Detroit, Michigan; 2 Henry Ford Hospital, Detroit, Michigan

## Abstract

**Background:**

Anti-spike monoclonal antibodies (mAb) including Bamlanivimab (BAM) and Bamlanivimab-Etesevimab (BAM/E) have shown reduced hospitalization rates for non-severe coronavirus disease 2019 (COVID-19) in clinical trials. Recent studies provided real-world hospitalization rates for BAM. But, similar data on those who received BAM/E are lacking. In spring 2021, Michigan experienced a surge of COVID-19 with more cases per capita than any other state. We sought to quantify the impact of BAM monotherapy versus BAM/E combination on hospitalization and mortality among a real-world high-risk cohort of outpatients with COVID-19.

**Methods:**

This retrospective cohort study evaluated outpatients ≥18 years with laboratory-confirmed mild/moderate COVID-19 who received mAb in a Detroit health system based on emergency use authorization criteria. Inclusion began on December 3^rd^ 2020 with BAM monotherapy, changed to BAM/E combination on March 27, 2021, and included patients until April 19^th^ 2021 (Figure 1). Demographics, comorbidities, and clinical characteristics were compared between patients who received BAM verses BAM/E using Chi-square and Mann-Whitney U test. Primary outcome was 30-day COVID-19 related hospitalization. Secondary outcomes were 30-day mortality and length of stay (LOS).

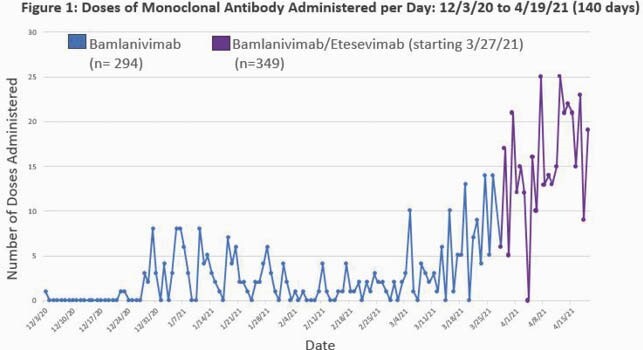

Inclusion began on December 3rd 2020 with BAM monotherapy, changed to BAM/E combination on March 27, 2021, and included patients until April 19th 2021. In spring 2021, Michigan experienced a surge of COVID-19 with more cases per capita than any other state resulting in a large sample of real-world patients for analysis.

**Results:**

643 patients received mAb (294 in BAM group and 349 in BAM/E group). Patients in the BAM/E cohort were younger and more obese with lower rates of diabetes, myocardial infarction, and cancer. Other characteristics were similar (Table 1). BAM/E patients had longer time from symptom onset to infusion (6 vs 4 days, p< 0.001). COVID-19 related 30-day hospitalization rates did not differ between groups (7.8 vs 7.2%, p=0.751). LOS and 30-day mortality (1% vs 0.3%, p=0.238) were also similar (Table 2).

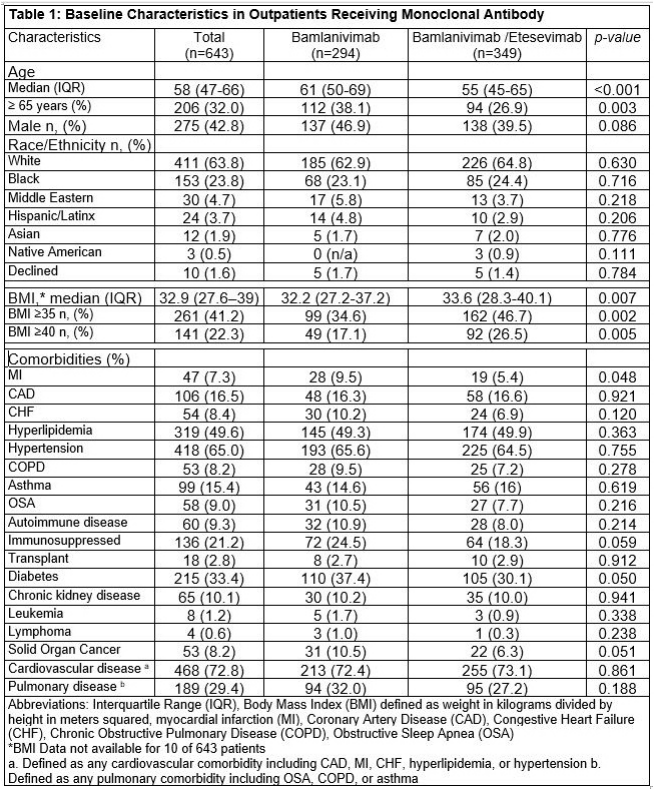

Patients in the BAM/E cohort were younger and more obese with lower rates of diabetes, myocardial infarction, and cancer. Other characteristics were similar

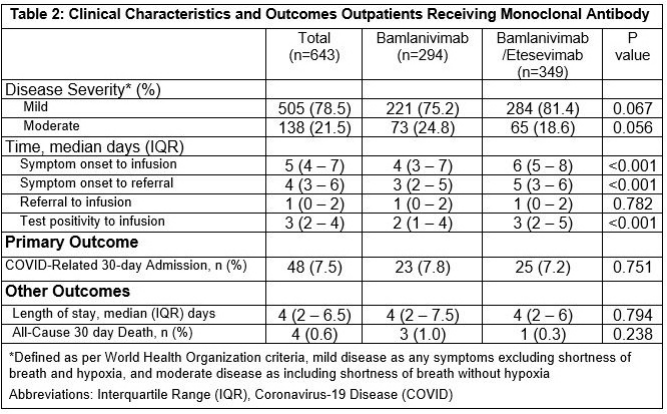

BAM/E patients had longer time from symptom onset to infusion (6 vs 4 days, p<0.001). COVID-19 related 30-day hospitalization rates did not differ between groups. Length of stay and 30-day mortality were also similar.

**Conclusion:**

Rates of hospitalization in our study were higher than in clinical trials of mAB and may reflect differences in study populations (Table 3). Compared to other real-world studies, our cohort of young, obese, and Black patients, had similar hospitalization rates of 7.5%. The lack of difference in outcomes noted among the mAB formulations in our study may be related to longer time from symptom onset to infusion in the BAM/E combination group.

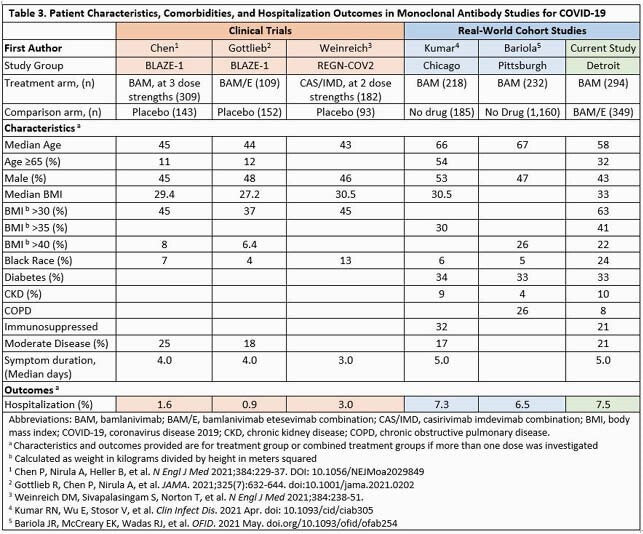

Our patients were older with higher rates of obesity and other comorbidities than those in clinical trials (shown in orange). Compared to other real-world studies (in blue), our cohort of younger, more obese Black patients had similar hospitalization rates of 7.5%.

**Disclosures:**

**All Authors**: No reported disclosures

